# A systematic review protocol for crime trends facilitated by synthetic biology

**DOI:** 10.1186/s13643-020-1284-1

**Published:** 2020-02-03

**Authors:** Mariam Elgabry, Darren Nesbeth, Shane D. Johnson

**Affiliations:** 1 DAWES Centre for Future Crime at UCL, Jill Dando Institute for Security and Crime Science, 35 Tavistock Square, London, WC1H 9EZ UK; 2 Department of Biochemical Engineering, University College London, Bernard Katz Building, London, WC1E 6BT UK

**Keywords:** Future crime, Biocrime, Systematic review, Emerging crime trends, Biotechnology, Methods

## Abstract

**Background:**

When new technologies are developed, it is common for their crime and security implications to be overlooked or given inadequate attention, which can lead to a ‘crime harvest’. Potential methods for the criminal exploitation of biotechnology need to be understood to assess their impact, evaluate current policies and interventions and inform the allocation of limited resources efficiently. Recent studies have illustrated some of the security implications of biotechnology, with outcomes of misuse ranging from compromised computers using malware stored in synthesised DNA, infringement of intellectual property on biological matter, synthesis of new threatening viruses, ‘genetic genocide,’ and the exploitation of food markets with genetically modified crops. However, there exists no synthesis of this information, and no formal quality assessment of the current evidence. This review therefore aims to establish what current and/or predicted crimes have been reported as a result of biotechnology.

**Methods:**

A systematic review will be conducted to identify relevant literature. ProQuest, Web of Science, MEDLINE and USENIX will be searched utilizing a predefined search string, and Backward and Forward searches. Grey literature will be identified by searching the official UK Government website (www.gov.uk) and the Global database of Dissertations and Theses. The review will be conducted by screening title/abstracts followed by full texts, utilising pre-defined inclusion and exclusion criteria. Papers will be managed using Eppi-center Reviewer 4 software, and data will be organised using a data extraction table using a descriptive coding tool. A predefined rating system (speculative, experimental or currently occurring) will be used to sort studies, and a thematic synthesis of the results will be presented.

**Discussion:**

Despite the concerns raised about the misuse of biotechnology, no previous work has been conducted from a Crime Science perspective to collate and assess the literature. This systematic review aims to identify the types of offending activity facilitated by biotechnology, including synthetic biology and genetic engineering. The objective of the review is to examine whether this offending activity can be prevented by assessing the conditions necessary for the crime events to occur. It is anticipated that evidence generated from this review will guide future research in this area and aid relevant stakeholders to prioritise and allocate limited resources to biotechnology crime prevention.

**Systematic review registration:**

PROSPERO CRD42019131685

## Background

The nature of crime is constantly evolving. In recent times, the advent of the internet has created a huge increase in crime opportunity, with around half of all crime now committed online [1, 2]. However, many other emerging technologies—such as biotechnology—may generate new crime opportunities. In fact, the UK Home Office has identified synthetic biology as an area that could pose future threats to national security [3], and the funds allocated to this issue by the UK Defence Science and Technology Laboratory (DSTL) have increased from £4 million in 2014 [4] to £45 million in 2019 [5].

The rationale behind the potential dual use of biotechnology dates back centuries to biological warfare and more recently to emerging technologies such as 3D printing [6, 7] and the Internet of Things (IoT) [8]. Biotechnology is here defined as per article 2 of the UN Convention on Biological Diversity, ‘any technological application that uses biological systems, living organisms or derivatives thereof, to make or modify products or processes for specific use’. This includes genetic engineering and synthetic biology techniques. Synthetic biology can be defined as an integrated subject area in which traditional biological systems are re-created or modified in novel ways for various application purposes, from medical diagnostics to environmental solutions. An example of the outcome of synthetic biology is biosensors designed to emit a signal in the presence of a signature disease characteristic or toxin [9]. This has turned what was considered a traditional biological field limited to laboratory tacit knowledge [10] into that of an engineering process [11], allowing for more rapid development and widespread application. As new technology matures, however, its misuse may be anticipated to increase too [12]. Misuse is defined as illegitimate activities that are punishable by law and as the exploitation of legitimate activities for criminal purposes. Examples of this in the context of biotechnology include compromising computers using malware stored in synthesised DNA [13], infringing intellectual property on the biological matter [14], synthesising threatening viruses [15], ‘genetic genocide’ [16] or exploiting food markets with genetically modified crops [17].

However, to date, there exists no synthesis of the varied malicious opportunities enabled or generated by biotechnology, either currently occurring or forecasted. Instead, researchers in the Life Sciences tend to focus on the benefits of these technologies for successful grant applications to further their research [18, 19], social scientists explore the ethical implications of the technology for society (e.g., eugenics [20–22]), and governmental officials highlight the exploitation potential for defence security applications [23].

This systematic review aims to extract reported studies that explore current and/or predicted crime facilitated by biotechnology, including synthetic biology and genetic engineering. Data analysis will be performed to synthesise evidence on (i) what forms of biotechnology have been shown to, or are expected to be, prone to criminal exploitation; (ii) what crime types have been discussed as already materialised; and (iii) what crime types are expected in the future. The outcomes of the review are intended to increase understanding of the risks and to identify policy (and other) implications for relevant (resource-limited) stakeholders to inform a biotechnology crime prevention agenda. Other implications include health and policy repercussions, for which interested stakeholders would include, but are not limited to, biotechnology researchers, scientific advisers for national security, policy makers and businesses.

## Methods/design

### Research question

What is the evidence on the forms of crime facilitated by biotechnology?

#### Objectives

To reveal evidence on (i) what forms of biotechnology have been shown to be prone to criminal exploitation, (ii) what crime types have been discussed as already materialised, (iii) what types of crime are expected in the future and (iv) what necessary conditions are for crime events to occur with a view to informing their prevention.

#### Study overview

**Fig. 1:**
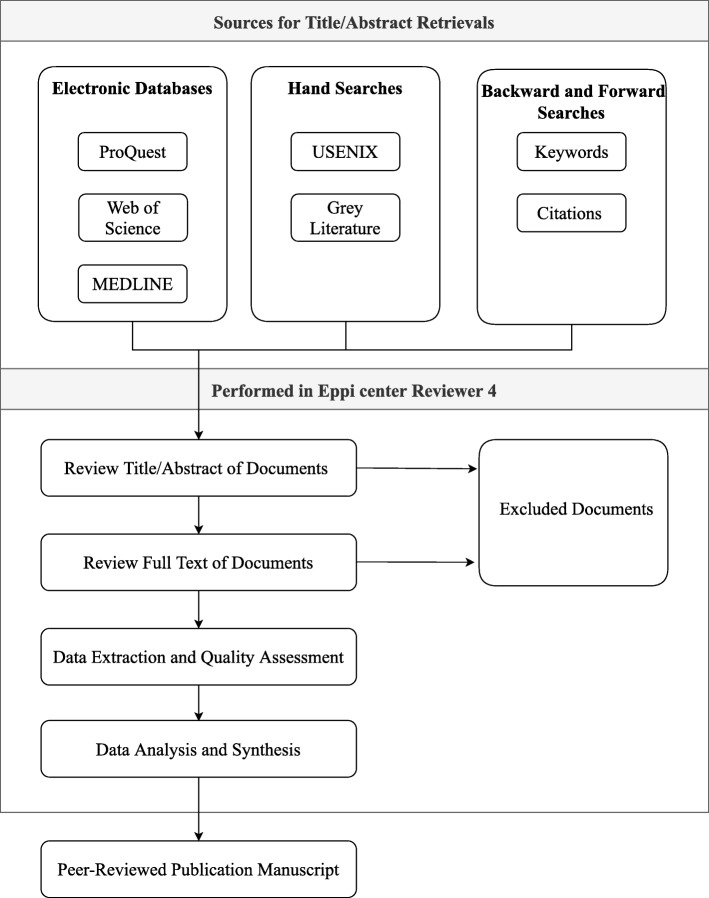
A flowchart of the study protocol

An overview of the study protocol is illustrated in a flow chart in Fig. 1.

To retrieve relevant academic studies, three electronic databases (ProQuest, Web of Science and MEDLINE) will be queried using a keyword search. An additional database, the Advanced Computing Systems Association (USENIX) will be hand searched. To retrieve relevant grey literature, the UK Government website and the Global database of Dissertations and Theses will be hand searched. Backward and Forward searches will be conducted to further identify relevant publications using keywords and citations of key papers. Forward searches refer to snowballing or finding (additional) studies that have cited the key studies identified through the initial search. Backward searches are used to identify past works that may be relevant by looking at the study reference lists of already identified articles [24–26]. To review and manage the retrieved studies, Eppi Center Reviewer software will be used. This is an online tool where references will be uploaded and analysed using its coding tools. A manuscript will be written upon completion of the data synthesis and submitted to a peer-reviewed journal.

#### Eligibility criteria

Table 1 details the inclusion and exclusion criteria organised according to a PICO format [27–29].

**Table 1:** A summary of the eligibility criteria for the screening phases of the systematic review

Criteria	Inclusion	Exclusion
Population(s)	Human	Animal, plant
Intervention(s)	Current or potential future misuse of biotechnology, synthetic biology and genetic engineering	Technology: medical devices Crime types: war crimes, crimes against humanity, intellectual Property and corporate liability crimes, agriculture and food security, wildlife/biodiversity crimes
Comparator	Not applicable	Not applicable
Outcomes	Scale of crime enabled by biotechnology Crime themes and sub-types Impacts of crime (health and policy) Individual/system-level characteristics of population/sector involved Contingency suggestions	The crime themes extracted will be synthesised for implications in the UK only.
Study design	Peer-reviewed, government document, or academic thesis only All study designs will be included. Each study will be ranked by our rating system hierarchically.	Commentaries Forewords Books/book reviews Articles Opinions Letters Editorials
Other	English language	Non-English

##### Types of studies

To be included, studies must be peer-reviewed, be an official government publication, a conference proceeding or a PhD thesis. Commentaries, book reviews and opinions will be excluded. The review will be limited to publications in the English language but from any country setting.

There are no restrictions on the types of study design eligible for inclusion. The studies will, however, be ranked hierarchically according to the study design, as follows:

1. Speculative (crime type has been suggested possible)
2. Experimental (crime type has been demonstrated through a proof-of-concept)
3. Currently occurring (crime type has been successfully implemented)

##### Types of participants

A broad approach will be taken so that we include studies that discuss how populations from the public, private and charity sector may be affected by the exploitation of biotechnology (from both the direct use of the technology but also the indirect impact). We will place no constraints on the age or gender of potential victims. Those affected may include, but are not limited to, biotechnology researchers, academics, regulatory bodies, healthcare providers, scientific advisers for national security, research councils, think tanks, policy makers, early adopters, consumers and businesses.

##### Conditions studied

Any reported studies that aim to illustrate the security implications of biotechnology will be considered in this review. This includes studies across synthetic biology and genetic engineering. Medical devices will be excluded, as there is a sufficient body of work focusing on the security implications of these devices, particularly from the perspective of cyber security [30–32]. Studies that are not relevant will be excluded from the review based on the following criteria:

1. They do not relate to a biotechnology, synthetic biology or genetic engineering tools, techniques or devices
2. They do not explicitly mention that these technologies can be a threat to person(s) in a community, have negative security implications, are/can be involved in crime or criminal exploitation or are/can be hacked

Studies that imply but do not explicitly articulate threats, risks or hazards will therefore not be included. An example of this would be if a study mentioned a disease threat in livestock of a modified virus but did not explicitly state how an attack would be implemented.

The study must consider current crimes facilitated by biotechnology or predicted future crime trends. All crime types will be included, except for war crimes. War crimes consist of bioweapons, bioterrorism and biodiversity impacts from agricultural attacks. As this review focuses on current and future crime trends, war crimes are excluded as their prohibition dates back to 1972 and have since been regulated by the following:

- Biological Weapons Convention entered into force (UN 2018a)
- United Nation Security Council Resolution 1540 (2004)
- International Health Regulations (IHR) (WHO 2008)
- Global Health Security Agenda (2018)

Studies related to food security will be excluded. Studies that relate to detection methods, such as forensic protocols applied to a crime scene, as opposed to focusing on the criminal activity facilitated by the technology will also be excluded. Likewise, studies that relate to emergency plans, mitigation or dissemination plans will be excluded. An example would be works that discuss emergency plans for accidental radiation exposure.

##### Outcomes measured

The primary outcomes measured in this review are as follows:

- The form and subsets of criminal opportunity in relation to the technology and whether it is biotechnology-dependent (types of crime that cannot otherwise be committed without the use of biotechnology) or biotechnology-enabled (traditional crime types which can be increased in their scale or reach by the use of biotechnology).
- The biotechnology sector and type most prone to such activity and characteristics that promote opportunity for offending.
- Individual-level and system-level characteristics of the necessary conditions for a biotechnology crime to occur (empirically substantiated risk factors or indicators could be used to target interventions).

The secondary outcomes measured in this review are as follows:

- The scale of identified and predicted biotechnology criminal cases and what proportion of the known biotechnology offending problem requires prioritisation.
- Health impacts of biotechnology crime on victims and related individuals (e.g. responses to the release of harmful pathogens as a result of penetrated DNA synthesisers).
- The jurisdictional implications of biotechnology crime within court (e.g. DNA bank databases compromised with false evidence).
- Contingency planning between public health authorities and other government bodies for dealing with biotechnology-related attacks intended to harm civilian populations.

If available, quantitative data, such as the cost of crime, and the scale of the problem will also be extracted.

#### Search strategy

The search strategy to retrieve relevant studies will involve a chain citation method (backward search) that in combination with snowballing (forward search) will identify articles that may have been missed through an automated search strategy [33, 34].

1. A pre-defined search query (specified below) that is constructed with key terms and applied to selected electronic databases.
2. Backward searches that will identify past works, which may be relevant, by looking at the reference lists of identified studies.
3. Forward searches that will find studies that have cited those identified through the initial search, for key studies only. The most cited studies from the identified articles will be selected as key studies.

#### Information sources

The following databases will be searched:

- ProQuest (Criminology Collection, Computer Science Database, Global Dissertations and Theses)
- Web of Science
- MEDLINE Ovid
- USENIX
- The official UK Government website (www.gov.uk)

#### Search queries and study selection

The search and retrieval of relevant studies involves examining databases that cover multiple disciplines (Life Science, Social Science and Computer Science sources). This requires careful consideration of biotechnology (1) and its exploitation (2) in the search string. These elements are described differently per discipline, an example being the use of the term ‘hacking’. In computer science, this means overcoming security barriers, while for biological publications, it refers to gaining a better understanding of biochemical processes within the Life Sciences [35, 36]. By searching the subject headings of articles (as well as keywords), the retrieval of articles will be based on the article *topic*. A search string on a subject heading will therefore retrieve related articles with that standardised word (rather than keyword). Subject headings may vary between databases; therefore, these will be searched first to identify the most relevant to the search query.

The keywords to be searched will include genetic engineering, synthetic biology, biotechnology, threat(s), threatening, crime(s), criminal(s), criminogenic, offend, offender(s), offending, secure, securing, security, hack(s), hacking, hacker(s). The search string to be used is shown below (note that this is formatted for use with ProQuest, but the same terms—reformatted—will be used for the other database searches):

(AB,TI(THREAT* OR CRIM* OR OFFEND* OR SECUR* OR HACK*)

*AND*

*SU.EXACT("genetic engineering" OR "SYNTHETIC BIOLOGY" OR "BIOTECHNOLOGY") )*

*OR*

(AB,TI((genetic NEAR/2 engineer*) OR (SYNTHETIC NEAR/2 BIOLOG*) OR BIOTECH*)

*AND*

*SU("THREAT" OR "CRIME" OR "OFFENDER" OR "SECURITY" OR "HACKERS" or "HACKING") )*

**Table:** 

*AB = abstract*	*TI = Title*	*SU = subject heading*

**Fig. 2:**
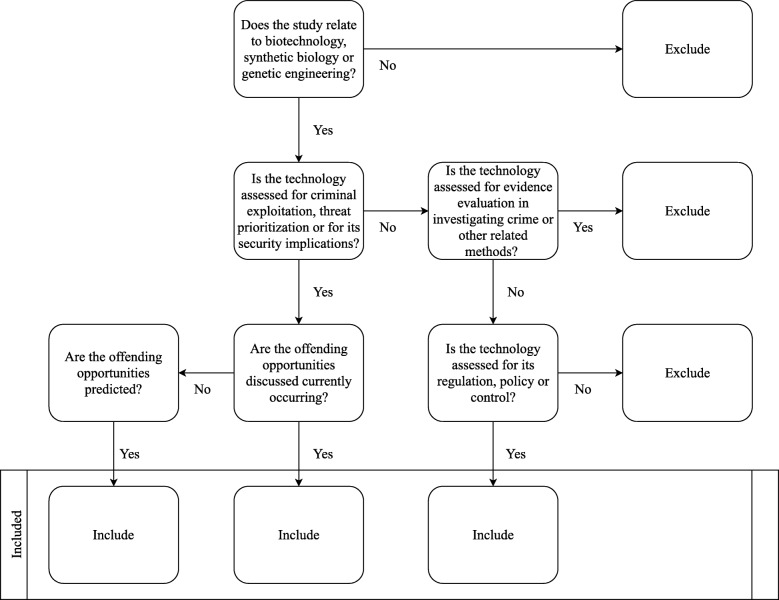
A diagram of the inclusion decision tree

The lead reviewer will review all titles, abstracts and full texts. Independent reviewers will perform a parallel review of the titles, abstracts and full texts, with each reviewer being assigned a percentage of the total retrieval items. Any discrepancies will be discussed and re-examined until an agreement is reached. The use of a tailored inclusion decision tree, Fig. 2, is predefined for the studies to maintain consistency and to help avoid coder drift. This includes control points on the context of the study.

#### Data extraction and quality assessment

The search criteria will be used to retrieve relevant studies. EndNote and Eppi-Center software [37] will be used as reference and management tools. Titles, abstracts and full texts identified will be screened by a single researcher against the predefined eligibility criteria. A random sample of these will then be screened by another two researchers across all stages of the review process. Coder drift [38] will be assessed through calculating the PABAK (prevalence-adjusted bias-adjusted kappa) statistic for inter-reliability as per [39], which takes into account the agreement that would be expected purely by chance.

A methodological difficulty within this study pertains in the assessment of the quality of the studies. It is difficult to assess the quality of the evidence of an event happening (i.e., crime type), when it has not happened yet (i.e., emerging crime type). A recently published systematic review shares this difficulty [40]. Moreover, it is expected that the extracted studies will consist of both quantitative and qualitative study designs. These will include a variety of methods that may involve laboratory experiments and also speculative interviews with experts. For the qualitative studies, there is no consensus in a suitable quality assessment framework [41, 42]. However, only peer-reviewed and PRISMA-compliant studies [43, 44] are selected in this review and can be used as a proxy for good quality. This review is conducted across multiple disciplines (Life sciences, Computer Science and Crime research) that may involve mixed methods, and so, traditional quality assessment tools such as those proposed by Campbell [45] cannot be applied sufficiently. As the purpose of this review is policy and practice-oriented, and where quantitative studies are extracted, the EMMIE framework [46] will be used. This brings together evidence of all dimensions of importance (not only measurements of effect size but also other influencing factors such as the mechanism in question) to inform policy and practice [46].

The extracted data will include study identifiers, study design descriptors, information on biotechnology in use and crime type details. A rating system and a section for notes will also be included (Table 2). The rating system will categorise each study hierarchically as either speculative, experimental or currently occurring. Speculative defines a study of crime types that have been suggested possible by experts. Experimental defines a study of crime types that have been demonstrated as possible through a proof-of-concept. Finally, currently occurring defines a study of crime types that have been successfully implemented.

**Table 2:** Data extraction coding summary table using Eppi-Center Reviewer 4

Data extraction coding	
Study identifiers	Search strategy, language, country, date published, keywords, study discipline
Design Description	Aims, methods, data sources, outcome measures
Content description	Biotechnology: sector, type, application, user, issue, investment, availability Crime: occurrence, attack type, offender type, crime setting
Study rating	Speculative Experimental Currently occurring
Notes	Other notes

#### Data synthesis

The thematic synthesis will be used as a qualitative method to identify and extract relevant themes (crime type) related to current and emerging offending trends in biotechnology. The aim is to synthesise crime types facilitated by biotechnologies that policy makers and others, with limited resources, might direct attention towards in terms of early detection and prevention.

The report produced for this review will be submitted as a paper to a leading journal in this field. Together with research that is conducted in parallel, the findings of this review are intended to guide potential changes in practice. These will be articulated through a knowledge transfer scheme to relevant stakeholders.

## Discussion

Implications of the potential misuse of biotechnology have been discussed previously by a number of authors and from a number of disciplinary perspectives [47, 48]. However, this has been done anecdotally without a systematic review of the literature. Here, we propose such a review to formally identify the current criminal opportunities and any emerging crime trends facilitated by this rapidly developing technology. As well as identifying trends, the review will assess the plausibility of particular offence types through an assessment of the extent to which the threats have been demonstrated as possible.

While systematic reviews are generally used to synthesise findings about existing or historic issues in an unbiased way, the approach has a substantial value in helping to identify and organise material about future issues [40], in this case crimes that might be facilitated by biotechnology, synthetic biology and genetic engineering. The aim of the review proposed here is to identify what is and what is not known about these issues to guide future research in this field and to identify potential policy implications. Doing so now provides the opportunity to inform policy in an anticipation of possible problems to avoid policy makers responding to them after they have emerged or escalated.

## Data Availability

Not applicable

## Abbreviations

PICO: Population, Intervention, Control/Comparison, Outcome
USENIX: Advanced Computing Systems Association
